# Raman spectroscopy of graphene under ultrafast laser excitation

**DOI:** 10.1038/s41467-017-02508-x

**Published:** 2018-01-22

**Authors:** C. Ferrante, A. Virga, L. Benfatto, M. Martinati, D. De Fazio, U. Sassi, C. Fasolato, A. K. Ott, P. Postorino, D. Yoon, G. Cerullo, F. Mauri, A. C. Ferrari, T. Scopigno

**Affiliations:** 1 Dipartimento di Fisica, Universitá di Roma La Sapienza”, Rome, I-00185 Italy; 2 Istituto Italiano di Tecnologia, Center for Life Nano Science @Sapienza, Rome, I-00161 Italy; 3 Institute for Complex Systems, CNR, UoS Sapienza, Rome, I-00185 Italy; 4 Cambridge Graphene Centre, University of Cambridge, Cambridge, CB3 OFA UK; 5 IFN-CNR, Dipartimento di Fisica, Politecnico di Milano, P.zza L. da Vinci 32, Milano, 20133 Italy

## Abstract

The equilibrium optical phonons of graphene are well characterized in terms of anharmonicity and electron–phonon interactions; however, their non-equilibrium properties in the presence of hot charge carriers are still not fully explored. Here we study the Raman spectrum of graphene under ultrafast laser excitation with 3 ps pulses, which trade off between impulsive stimulation and spectral resolution. We localize energy into hot carriers, generating non-equilibrium temperatures in the ~1700–3100 K range, far exceeding that of the phonon bath, while simultaneously detecting the Raman response. The linewidths of both G and 2D peaks show an increase as function of the electronic temperature. We explain this as a result of the Dirac cones’ broadening and electron–phonon scattering in the highly excited transient regime, important for the emerging field of graphene-based photonics and optoelectronics.

## Abstract (web summary)

Non-equilibrium ultrafast processes in graphene entail relaxation pathways involving electron–electron and electron–phonon scattering events. Here, the authors probe graphene optical phonons at high electronic temperatures by means of Raman spectroscopy under pulsed excitation

## Introduction

The distribution of charge carriers has a pivotal role in determining fundamental features of condensed matter systems, such as mobility, electrical conductivity, spin-related effects, transport, and optical properties. Understanding how these proprieties can be affected and, ultimately, manipulated by external perturbations is important for technological applications in diverse areas, ranging from electronics to spintronics, optoelectronics and photonics^1–3^.

The current picture of ultrafast light interaction with single-layer graphene (SLG) can be summarized as follows^4^. Absorbed photons create optically excited electron–hole (e–h) pairs. The subsequent relaxation towards thermal equilibrium occurs in three steps. Ultrafast electron–electron (e–e) scattering generates a hot Fermi–Dirac distribution within the first tens fs^5^. The distribution then relaxes due to scattering with optical phonons (ph; electron–phonon (e–ph) coupling), equilibrating within a few hundred fs^6,7^. Finally, anharmonic decay into acoustic modes establishes thermodynamic equilibrium on the ps timescale^8–10^.

Raman spectroscopy is one of the most used characterization techniques in carbon science and technology^11^. The measurement of the Raman spectrum of graphene^12^ triggered a substantial effort to understand phonons, e–ph, magneto–ph, and e–e interactions in graphene, as well as the influence of the number and orientation of layers, electric or magnetic fields, strain, doping, disorder, quality and types of edges, and functional groups^13–15^. The Raman spectra of SLG and few layer graphene (FLG) consist of two fundamentally different sets of peaks. Those, such as D, G, 2D, present also in SLG, and due to in-plane vibrations^12^, and others, such as the shear (C) modes^16^ and the layer breathing modes^17,18^ due to relative motions of the planes themselves, either perpendicular or parallel to their normal. The G peak corresponds to the high frequency *E*_2g_ phonon at Γ. The D peak is due to the breathing modes of six-atom rings, and requires a defect for  its activation^19–21^. It originates from transverse optical (TO) phonons around the Brillouin Zone edge **K**[19], it is active by double resonance (DR)[20] and, due to a Kohn Anomaly at **K**[22], it is dispersive with excitation energy. The 2D peak is the D overtone and originates from a process where momentum conservation is fulfilled by two phonons with opposite wavevectors. It is always present since no defects are required for this process^12^.

Raman spectroscopy is usually performed under continuous wave (CW) excitation, therefore probing samples in thermodynamic equilibrium. The fast e–e and e–ph non-radiative recombination channels establish equilibrium conditions between charge carriers and lattice, preventing the study of the vibrational response in presence of an hot e–h population. Using an average power comparable to CW illumination (a few mW), ultrafast optical excitation can provide large fluences (~1−15 J m^−2^ at MHz repetition rates) over sufficiently short timescales (0.1–10 ps) to impulsively generate a strongly out-of-equilibrium distributions of hot e–h pairs^4,8,23,24^. The potential implications of coupled e and ph dynamics for optoelectronics were discussed for nanoelectronic devices based on CW excitation^25–29^. However, understanding the impact of transient photoinduced carrier temperatures on the colder SLG ph bath is important for mastering out of equilibrium e–ph scattering, critical for photonics applications driven by carrier relaxation, such as ultrafast lasers^30^, detectors^1,3^ and modulators^31^. E.g, SLG can be used as a much broader-band alternative to semiconductors saturable absorbers^30^, for mode-locking and Q switching^1,30^.

Here we characterize the SLG optical ph at high electronic temperature, *T*_e_, by performing Raman spectroscopy under pulsed excitation. We use a 3 ps pulse to achieve a trade-off between the narrow excitation bandwidth required for spectral resolution $\left( {\frac{{\delta \nu }}{c} \le } \right.$ 10 cm^−1^, being *v*[Hz] the laser frequency and c the speed of light, a condition met under CW excitation) and a pulse duration, *δt*, sufficiently short (*δt* ≤ 10 ps, achieved using ultrafast laser sources) to generate an highly excited carrier distribution over the equilibrium ph population, being those two quantities Fourier conjugates^32^ $\left( {\frac{{\delta \nu }}{{\delta t}} \le 14.7{\mathrm{cm}}^{ - 1}\mathrm{ps}} \right)$. This allows us to determine the dependence of both ph frequency and dephasing time on *T*_e_, which we explain by a broadening of the Dirac cones.

## Results

### Hot photoluminescence

**Fig. 1:**
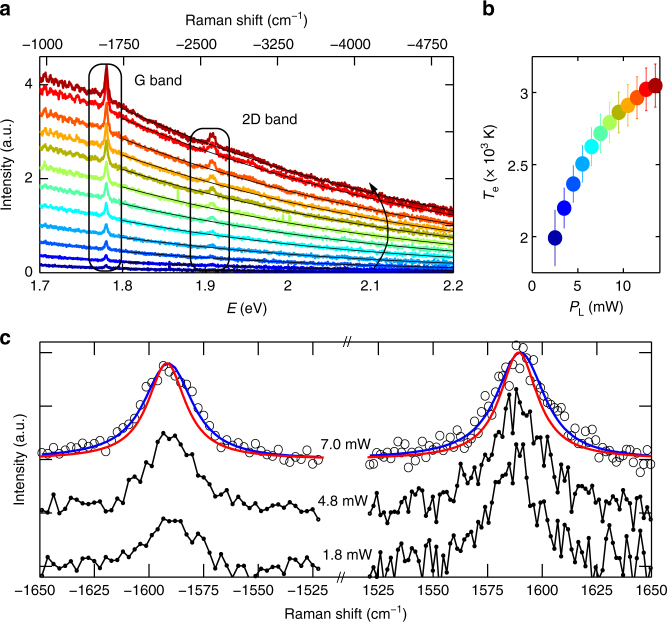
Spectral response of SLG under ultrafast excitation. **a** AS Raman spectra under ultrafast excitation for laser powers increasing along the arrow direction. The *P*_L_-dependent background is fitted by thermal emission (Eq. 1, black lines) resulting in *T*_e_ in the 1700–3100K range. **b** *T*_e_ as a function of P_*L*_. Error bars represent the 95% confidence bounds of the best fit. **c** Background subtracted, AS and S G peak (in black, normalized to the corresponding S maximum) measured as function of *P*_L_ in the range $\sim 1.8 \div 7.0$ mW (corresponding to $T_{\mathrm{e}} \sim 2000 \div 2700$K). Three representative *P*_L_ values are shown. Best fits of the G peak (blue line), obtained as a convolution of a Lorentzian (red line) with the IRF are also reported for the largest *P*_L_ value

Figure 1a plots a sequence of anti-Stokes (AS) Raman spectra of SLG following ultrafast excitation at 1.58 eV, as a function of excitation power *P*_L_. The broad background stems from hot photoluminescence (PL) due to the inhibition of a full non-radiative recombination under high excitation densities^8,26,33,34^. This process, absent under CW excitation in pristine SLG^35^, is due to ultrafast photogeneration of charge carriers in the conduction band, congesting the e–ph decay pathway, which becomes progressively less efficient with increasing fluence. This non-equilibrium PL recalls the gray body emission and can be in first approximation described by Planck’s law:^8,26,29,33^

1 $$I(\hbar \omega ,T_e) = {\cal R}(\hbar \omega )\tau _{{\mathrm{em}}}\eta \frac{{\hbar \omega ^3}}{{2\pi ^2c^2}}\left( {e^{\frac{{\hbar \omega }}{{kT_e}}} - 1} \right)^{ - 1}$$

where *η* is the emissivity, defined as the dimensionless ratio of the thermal radiation of the material to the radiation from an ideal black surface at the same temperature as given by the Stefan–Boltzmann law^36^, *τ*_em_ is the emission time and ${\cal R}(\hbar \omega )$ is the frequency dependent, dimensionless responsivity of our detection chain. Refs^8,29,33^ reported that, although Eq. 1 does not perfectly reproduce the entire gray body emission, the good agreement on a ~0.5 eV energy window is sufficient to extract *T*_e_. By fitting the background of the Raman spectra with Eq. 1 (solid lines in Fig. 1a) we obtain *T*_e_ as a function of *P*_L_. Figure 1b shows that *T*_e_ can reach up to 3100 K under our pulsed excitation conditions.

An upper estimate for the lattice temperature, *T*_1_, can be derived assuming a full thermalization of the optical energy between vibrational and electronic degrees of freedom, i.e., evaluating the local equilibrium temperature, *T*_eq_, by a specific heat argument (see Methods). We get *T*_eq_(*P*_max_) ~ 680 K at the maximum excitation power, *P*_max_ = 13.5 mW. This is well below the corresponding *T*_e_, indicating an out-of-equilibrium distribution of charge carriers. Thus, over our 3 ps observation timescale, *T*_1_ is well below *T*_eq_.

### Out of equilibrium Raman response

**Fig. 2:**
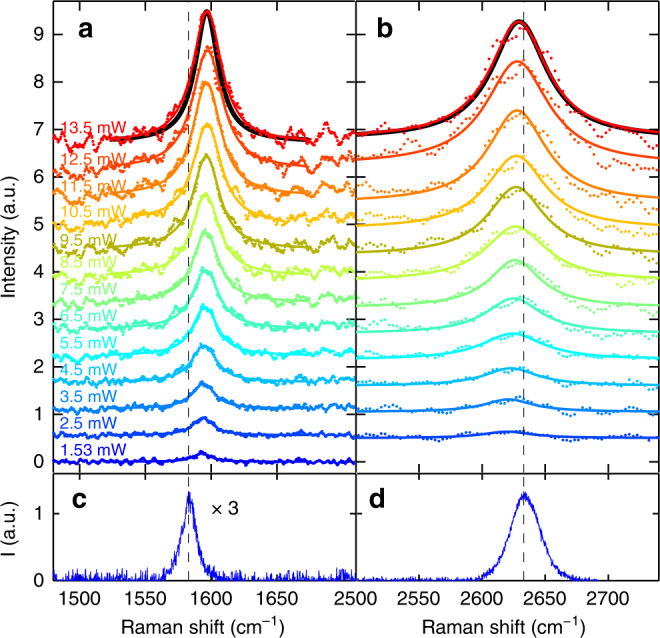
Raman spectra at different laser power. **a** AS G and **b** 2D peak as function of *P*_L_. (dots) Experimental data. (Lines) fitted Lorentzians convoluted with the spectral profile of the excitation pulse. The vertical dashed lines are the equilibrium, RT, Pos(G) and Pos(2D). **c** RT CW S G and **d** 2D peaks. The CW 2D is shifted by 5.4 cm^−1^ for comparison with the AS ps-Raman, see Methods. The relative calibration accuracy is ∼2 cm^−1^

**Fig. 3:**
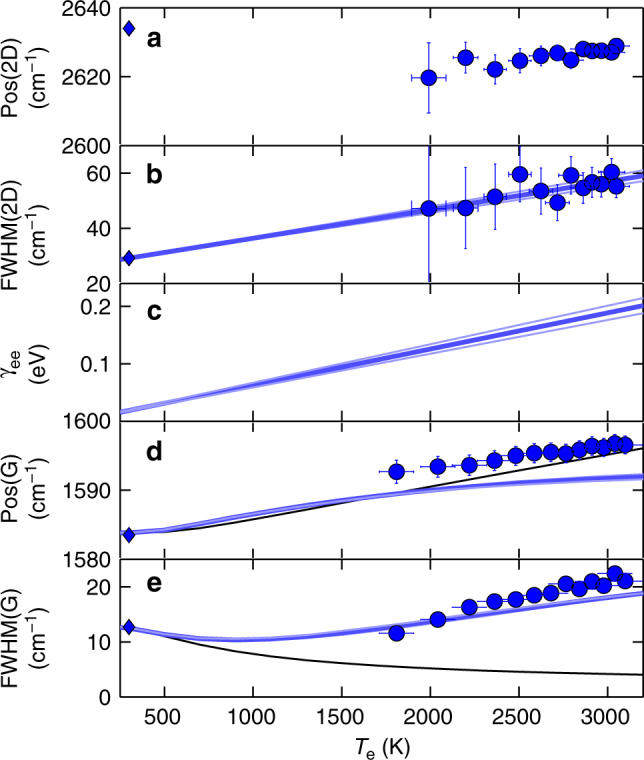
Comparison between theory and experiments. **a** Pos(2D), **b** FWHM(2D), **d** Pos(G), **e** FWHM(G) as a function of *T*_e_ for ps-excited Raman spectra. Error bars represent the 95% confidence level in the best fitting procedure. Solid diamonds in a,b,d,e represent the corresponding CW measurements. FWHM(2D) are used to determine the e–e contribution (*γ*_ee_) to the Dirac cones broadening, shown in **c** (blue lines). Pos(G) and FWHM(G) are compared with theoretical predictions accounting for e–ph interaction in presence of electronic broadening (an additional RT anharmonic damping ∼2 cm^−1^ ^10^ is included in the calculated FWHM(G)). Black lines are the theoretical predictions for *γ*_ee_ = 0 eV, while blue lines take into account an electronic band broadening linearly proportional to *T*_e_ (*γ*_ee_ = *α*_*e*_*k*_B_*T*_e_). From the fit of *γ*_ee_ in **c**, we get $\frac{{\alpha _ek_{\rm B}}}{{hc}}$ = 0.51 cm^−1^K^−1^ (thickest blue line). Values of $\frac{{\alpha _{{e}}k_{\rm B}}}{{hc}}$ = 0.46,0.55 cm^−1^K^−1^, corresponding to 99% confidence boundaries, are also shown (thin light blue lines)

Figure 1c plots the AS and S G peaks, together with fits by Lorentzians (blue lines) convoluted with the laser bandwidth (~9.5 cm^−1^) and spectrometer resolution (~6 cm^−1^), which determine the instrumental response function, IRF (see Methods). The S data have a larger noise due to a more critical background subtraction, which also requires a wider accessible spectral range (see Methods). For this reason, we will focus on the AS spectral region, with an higher spectrometer resolution (1.2 cm^−1^), Fig. 2. We obtain a full width at half maximum of the G peak, FWHM(G) ~21 cm^−1^, larger than the CW one (~12.7 cm^−1^). Similarly, we get FWHM(2D) ~50–60 cm^−1^ over our *P*_L_ range, instead of FWHM(2D) ~29 cm^−1^ as measured on the same sample under CW excitation. To understand the origin of such large FWHM(G) andFWHM(2D) in pulsed excitation, we first consider the excitation power dependence of the SLG Raman response in the 1.53–13.5 mW range (the lower bound is defined by the detection capability of our setup). This shows that the position of the G peak, Pos(G), is significantly blueshifted (as reported for graphite in ref^23^.), while the position of the 2D peak, Pos(2D), is close to that measured under CW excitation, and both FWHM(G) and FWHM(2D) increase with *P*_L_. Performing the same experiment on Si proves that the observed peaks broadening is not limited by our IRF (see Methods). Moreover, even the low resolution S data of the G band, collected in the range 1.8–7.0 mW (a selection is shown in Fig. 1c), display a broadening ((8 ± 4)10^−3^ cm^−1^ K^−1^) and upshift ((2.8 ± 1.8)10^−3^ cm^−1^ K^−1^), compatible with that of the high-resolution AS measurements (Fig. 3d, e) (7.4 ± 0.5)10^−3^ cm^−1^ K^−1^ and (3.2 ± 0.2)10^−3^ cm^−1^ K^−1^.

We note that phonons temperature estimates based on the AS/S intensity ratio^37,38^  are not possible in our case due to two concurring effects. First, SLG’s resonant response to any optical wavelength gives a non trivial wavelength dependent Raman excitation profile, which modifies the Raman intensities with respect to the non-resonant case. Consequently, the AS/S ratio is no longer straightforwardly related to the thermal occupation^39^. Second, in SLG a S ph may be subsequently annihilated by a correlated AS event.  This may result into an extra AS pumping which does not allow to relate AS/S ratio and ph temperature via the thermal occupation factor^40^. Accordingly, the AS/S ratio approaching one at the largest excitation power in Fig. 1c (black circles) does not necessary imply a large increase of the G phonon temperature.

## Discussion

**Fig. 4:**
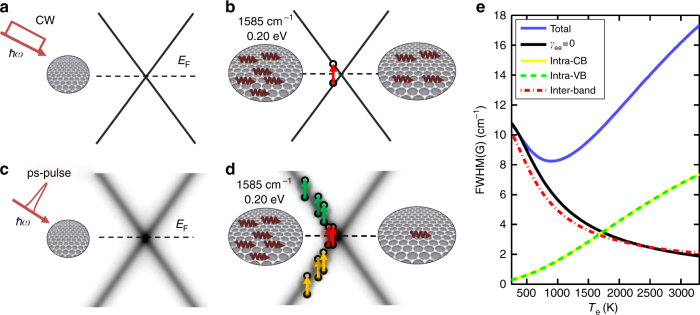
Effect of Dirac cone broadening on Raman process. **a** CW photoexcitation with mW power does not affect the Dirac cone. **b** Accordingly, e–h formation induced by e–ph scattering only occurs in presence of resonant ph excitation. **c** Under ps excitation, with average *P*_L_ comparable to **a**, the linear dispersion is smeared by the large *k*_B_*T*_e_ ≈ *ħω*_G_ = 0.2 eV. **d** Consequently, e–h formation is enhanced by the increased ph absorption cross section, due to new intra-band processes. **e** Corresponding contributions to FWHM(G) for the broadened inter-bands and intra-band processes for *α*_e_*k*_B_ = 0.51 cm^−1^ K^−1^

Figure 3 plots Pos(2D), FWHM(2D), Pos(G), FWHM(G) as a function of *T*_e_, estimated from the hot-PL. A comparison with CW measurements (633 nm) at room temperature (RT) is also shown (blue diamonds). Under thermodynamic equilibrium, the temperature dependence of the Raman spectrum of SLG is dominated by anharmonicity, which is responsible for mode softening, leading to a redshift of the Raman peaks^10,41,42^. This differs from our experiments (Fig. 4a–d), in which Pos(G) has an opposite trend (blue shift), and Pos(2D) is nearly *T*_e_ independent, in agreement with density functional perturbation theory (DFPT) calculations, giving a variation of the 2D peak ΔPos(2D) ~5 cm^−1^ in the range *T*_e_ = 300–3000 K (see Methods). This indicates the lack of significant anharmonic effects and suggests a dominant role of e–ph interaction on FWHM(G) and Pos(G), in the presence of a cold phonon bath at constant *T*_1_ decoupled from the (large) *T*_e_.

To derive the temperature dependence of such parameters, we first compute the phonon self-energy ${\Pi}(q = 0,\omega _{\mathrm{G}}^0)$, as for refs.^22,43,44^,

$${\Pi}(q = 0,\omega _G^0,T_{\mathrm {e}}) = \xi {\int}_0^{\tilde \varepsilon } {\mathrm {d}}\varepsilon \varepsilon {\int}_{ - \infty }^{ + \infty } {\mathrm{d}}z {\mathrm {d}}z{\prime}\mathop {\sum}\limits_{s,s{\prime}}$$

2 $$M_s(z,\varepsilon )M_{s{\prime}}(z{\prime},\varepsilon )\left[\frac{{f_{\mathrm{F}}(z - E_{\mathrm{F}}) - f_{\mathrm{F}}(z{\prime} - E_{\mathrm{F}})}}{{z - z{\prime} - \hbar \omega _{\mathrm{G}}^0 + {\mathrm{i}}\delta }}\right]$$

Here $\xi = g^2/(2\hbar m_{\mathrm{a}}\omega _{\mathrm{G}}^0v_{\mathrm{F}}^2) = 4.43 \times 10^{ - 3}$ is a dimensionless constant, *v*_F_ is the Fermi velocity, $\tilde \varepsilon$ is the upper cutoff of the linear dispersion *ε* = *v*_F_|***k***|, *m*_a_ is the carbon atom mass, $\hbar \omega _{\mathrm{G}}^0 = 0.196$eV the bare phonon energy*, δ* is a positive arbitrary small number (< 4 meV), *g* ~ 12.3 eV is proportional to the e–ph coupling^6,22,43,45^, *z*, *z*′ are the energy integration variables and *f*_F_(*z* − *E*_F_) is the Fermi–Dirac distribution with *E*_F_ the Fermi energy. Although our samples are doped, *E*_F_ significantly decreases as a function of *T*_e_^25^. Hence, we assume *E*_F_ = 0 in the following calculations. The two indexes *s*,*s*′ = $\mp 1$ denote the e and h branches, and *M*_s_(*z*, *ε*) is the corresponding spectral function, which describes the electronic dispersion.

The self-energy expressed by Eq. 2 renormalizes the phonon Green’s function according to the Dyson’s equation:^46^

3 $$D(\omega ) = \frac{{2\hbar \omega _{\mathrm{G}}^0}}{{(\hbar \omega + i\delta )^2 - (\hbar \omega _{\mathrm{G}}^0)^2 - 2\hbar \omega _{\mathrm{G}}^0{\Pi}(\omega )}}$$

so that ΔPos(G) and FWHM(G) can be written as:

4 $$\begin{array}{*{20}{l}} {\Delta {\mathrm {POS}}({\mathrm{G}}}) \hfill & { = \frac{1}{{hc}}{\mathrm{Re}}\left[ {{\Pi}(0,\omega _{\mathrm{G}}^0,T_{\mathrm{e}}) - {\Pi}(0,\omega _{\mathrm{G}}^0,T_{\mathrm{e}} = 0)} \right]} \hfill \\ {{{\mathrm {FWHM}}({\mathrm{G}})}} \hfill & { = - \frac{2}{{hc}}{\mathrm{Im}}{\Pi}(0,\omega _{\mathrm{G}}^0,T_{\mathrm{e}})} \hfill \end{array}$$

where *h* is the Planck constant. FWHM(G) can be further simplified since the evaluation of ${\mathrm{Im}}{\Pi}(0,\omega _{\mathrm{G}}^0,T_{\mathrm{e}})$ leads to $\delta (z - z{\prime} - \hbar \omega _{\mathrm{G}}^0)$ in Eq. 2, so that we get:

$${{\mathrm{FWHM}}({\mathrm{G}})} = \frac{{\pi \xi }}{{hc}}{\int}_0^{\tilde \varepsilon } {\mathrm{d}}\varepsilon \varepsilon {\int}_{ - \infty }^{ + \infty } {\mathrm{d}}z\mathop {\sum}\limits_{s,s{\prime}}$$

5 $$M_s(z,\varepsilon )M_{s{\prime}}(z - \hbar \omega _{\mathrm{G}}^0,\varepsilon )[f_{\mathrm{F}}(z) - f_{\mathrm{F}}(z - \hbar \omega _{\mathrm{G}}^0)]$$

In the limit of vanishing broadening of the quasiparticle state, the SLG gapless linear dispersion is represented by the following spectral function:^46^

6 $$M_s(z,\varepsilon ) = \delta (z + s\varepsilon ),\quad s = \pm 1,$$

This rules the energy conservation in Eq. 5 and allows only transitions between h and e states with energy difference $2\varepsilon = \hbar \omega _{\mathrm{G}}^0$. Thus, we get:^22,43,44^

7 $${{\mathrm{FWHM}}({\mathrm{G}})} = {{\mathrm{FWHM}}({\mathrm{G}})}^0\left[ {f_{\mathrm{F}}( - \hbar \omega _{\mathrm{G}}^0/2) - f_{\mathrm{F}}(\hbar \omega _{\mathrm{G}}^0/2)} \right]$$

where ${{\mathrm{FWHM}}}{({\mathrm{G}})}^0 = \frac{{\pi \xi \hbar \omega _{\mathrm{G}}^0}}{{2hc}} \sim 11$cm^−1^ ^10^. This value, with the additional ~2 cm^−1^ contribution arising from anharmonic effects^10^, is in agreement with the CW measurements at *T*_e_ = *T*_eq_ = 300 K (see diamond in Fig. 3e), corresponding to fluences ≪1 J m^−2^. Eq. 7 also shows that, as *T*_*e*_ increases, the conduction band becomes increasingly populated, making the phonon decay channel related to e–h formation progressively less efficient and leading to an increase of the ph decay time (Fig. 4b). This leads to a decrease of FWHM(G) for increasing *T*_e_ (black solid line in Fig. 3e), in contrast with the experimentally observed increase (blue circles in Fig. 3e).

A more realistic description may be obtained by accounting for the effect of *T*_e_ on the energy broadening (*γ*_e_) of the linear dispersion *M*_s_(*z*,*ε*), along with the smearing of the Fermi function. *γ*_e_(*z*,*T*_e_) can be expressed, to a first approximation, as the sum of three terms:^47^

8 $$\gamma _{\mathrm{e}}(z,T_{\mathrm{e}}) = \gamma _{{\mathrm{ee}}}(T_{\mathrm{e}}) + \gamma _{{\mathrm{ep}}}(z) + \gamma _{{{\mathrm{def}}}}(z)$$

where *γ*_ee_, *γ*_ep_ and *γ*_def_ are the e–e, e–ph and defect contributions to *γ*_e_. The only term that significantly depends on *T*_e_ is *γ*_ee_, while the others depend on the energy *z*^10,44,47–50^.

The linear dependence of *γ*_ee_ on *T*_e_^51^ can be estimated from its impact on FWHM(2D). The variation of FWHM(2D) with respect to RT can be written as:^42^

9 $$\Delta {{\mathrm{FWHM}}(2{\mathrm{D}})} = 4\sqrt {2^{2/3} - 1} \frac{1}{2}\frac{{\partial {{\mathrm{POS}}(2{\mathrm{D}})}}}{{\partial (h\nu _{{\mathrm{laser}}})}}\gamma _{{\mathrm{ee}}}$$

where $[\partial {{\mathrm{POS}}(2{\mathrm{D}})}/\partial (h\nu _{{\mathrm{laser}}})]/2 = \frac{1}{{ch}}v_{\mathrm{ph}}/v_{\mathrm{F}}{\mathrm{\sim }}100$cm^−1^ eV^−1^^13,52^, i.e., the ratio between the ph and Fermi velocity, defined as the slope of the phononic (electronic) dispersion at the ph (e) momentum corresponding to a given excitation laser energy *hν*_laser_^13^. Since the DR process responsible for the 2D peak involves the creation of e–h pairs at energy $\mp$ *hν*_laser_/2, the change of FWHM(2D) allows us to estimate the variation of *γ*_e_ at $z = h\nu _{{\mathrm{laser}}}/2 \simeq 0.8$eV. Then, *γ*_ep_ and *γ*_def_, both proportional to *z* (*γ*_ep_,*γ*_def_ ∝ *z*), will give an additional constant contribution to FWHM(2D), but not to its variation with *T*_e_. Our data support the predicted^51^ linear increase of *γ*_ee_ with *T*_e_, with a dimensionless experimental slope $\alpha _e \simeq 0.73$, Fig. 3c.

In order to compute FWHM(G) from Eq. 2, we note that the terms *γ*_ep_ and *γ*_def_ are negligible at the relevant low energy $z = \hbar \omega _{\mathrm{G}}/2 \sim 0.1$eV $\ll h\nu _{{\mathrm{laser}}}/2$. Hence $\gamma _{\mathrm{e}}(z,T_{\mathrm{e}}) \simeq \gamma _{{\mathrm{ee}}}(T_{\mathrm{e}})$.

The Dirac cone broadening can now be introduced by accounting for *γ*_*e*_ in the spectral function of Eq. 6:

10 $$M_{\mathrm{s}}(z) = \frac{1}{\pi }\frac{{\gamma _{\mathrm{e}}/2}}{{(z + s\varepsilon )^2 + (\gamma _{\mathrm{e}}/2)^2}},\quad s = \pm 1,$$

accordingly, all the processes where the energy difference |*sε*(*k*) − *s*′*ε*(*k*′) + *ħω*_0_| is less than 2*γ*_e_ (which guarantees the overlap between the spectral functions of the quasiparticles) will now contribute in Eq. 2. Amongst them, those transitions within the same (valence or conduction) band, as shown in Fig. 4d.

The broadened interband contributions still follow, approximately, Eq. 7 (Fig. 4e). However, the Dirac cone broadening gives additional channels for G phonon annihilation by carrier excitation. In particular, intra-band transitions within the Dirac cone are now progressively enabled for increasing *T*_e_, as sketched in Fig. 4d. In Fig. 4e the corresponding contributions to FWHM(G) are shown. Calculations in the weak-coupling limit^51^ suggest that *γ*_e_(*T*_e_) should be suppressed as *z* → 0, due to phase–space restriction of the Dirac cone dispersion. Our results, however, indicate that this effect should appear at an energy scale smaller than ℏ*ω*_G_/2, as the theory captures the main experimental trends, just based on a *z*-independent *γ*_e_(*T*_e_).

Critically, the G peak broadening has a different origin from the equilibrium case^53^. The absence of anharmonicity would imply a FWHM(G) decrease with temperature due to the e–ph mechanism. However, the Dirac cone broadening reverses this trend into a linewidth broadening above *T*_e_ = 1000K producing, in turn, a dephasing time reduction, corresponding to the experimentally observed FWHM(G) increase. The blue shift of the G peak with temperature is captured by the standard e–ph interaction, beyond possible calibration accuracy. Importantly, the Dirac cone broadening does not significantly affect Pos(G).

In conclusion, we measured the Raman spectrum of SLG with impulsive excitation, in the presence of a distribution of hot charge carriers. Our excitation bandwidth enables us to combine frequency resolution, required to observe the Raman spectra, with short pulse duration, needed to create a significant population of hot carriers. We show that, under these strongly non-equilibrium conditions, the Raman spectrum of graphene cannot be understood based on the standard low fluence picture, and we provide the experimental demonstration of a broadening of the electronic linear dispersion induced by the highly excited carriers. Our results shed light on a peculiar regime of non-equilibrium Raman response, whereby the e–ph interaction is enhanced. This has implications for the understanding of transient charge carrier mobility under photoexcitation, important to study SLG-based optoelectronic and photonic devices^27,28^, such as broadband light emitters^29^, transistors and optical gain media^54^.

## Methods

### Sample preparation and CW raman characterization

**Fig. 5:**
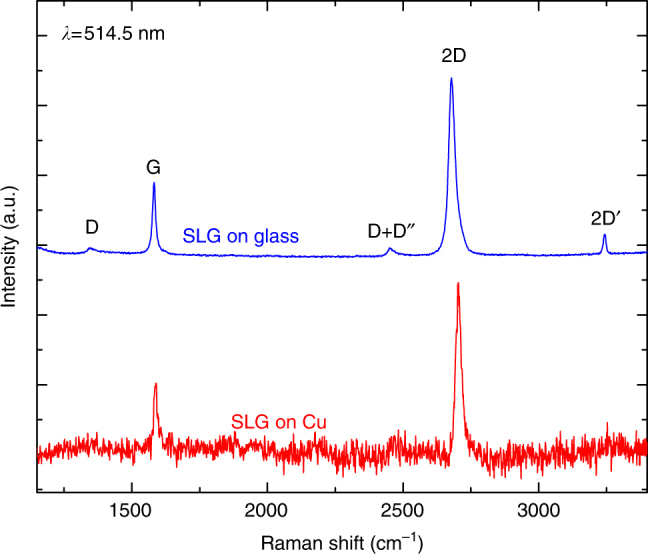
CW Raman spectra of SLG. Raman response of SLG on Cu (red line), and on glass (blue line) after the transfer from Cu. In the latter case, the substrate spectrum is subtracted

SLG is grown on a 35 μm Cu foil, following the process described in Refs^55,56^. The substrate is heated to 1000 °C and annealed in hydrogen (H_2_, 20 sccm) for 30 min. Then, 5 sccm of methane (CH_4_) is let into the chamber for the following 30 min so that the growth can take place^55,56^. The sample is then cooled back to RT in vacuum (∼1 mTorr) and unloaded from the chamber. The sample is characterized by CW Raman spectroscopy using a Renishaw inVia Spectrometer equipped with a 100× objective. The Raman spectrum measured at 514 nm is shown in Fig. 5 (red curve). This is obtained by removing the non-flat background Cu PL^57^. The absence of a significant D peak implies negligible defects^12,13,21,58^. The 2D peak is a single sharp Lorentzian with FWHM(2D) ∼23 cm^−1^, a signature of SLG^12^. Pos(G) is ∼1587 cm^−1^, with FWHM(G) ∼14 cm^−1^. Pos(2D) is ∼2705 cm^−1^, while the 2D to G peak area ratio is ∼4.3. SLG is then transferred on glass by a wet method^59^. Poly-methyl methacrylate (PMMA) is spin coated on the substrate, which is then placed in a solution of ammonium persulfate (APS) and deionized water. Cu is etched^55,59^, the PMMA membrane with attached SLG is then moved to a beaker with deionized water to remove APS residuals. The membrane is subsequently lifted with the target substrate. After drying, PMMA is removed in acetone leaving SLG on glass. The SLG quality is also monitored after transfer. The Raman spectrum of the substrate shows features in the D and G peak range, convoluted with the spectrum of SLG on glass (blue curve in Fig. 5). A point-to-point subtraction is needed to reveal the SLG features. After transfer, the D peak is still negligible, demonstrating that no significant additional defects are induced by the transfer process. The fitted Raman parameters indicate p doping ∼250 meV^50,60^.

Before and after the pulsed laser experiments, equilibrium CW measurements are performed at RT using a micro-Raman setup (LabRAM Infinity). A different energy and momentum of the D phonon is involved, for a given excitation wavelength, in the S or AS processes, due to the phonon dispersion in the DR mechanism^61,62^. Hence, in order to measure the same D phonon in S and AS, different laser excitations (*ν*_laser_) must be used according to $\nu_{\mathrm{laser}}^{\rm S} = \nu _{{\rm laser}}^{{\rm AS}} + {\rm cPos}(2{\mathrm{D}})$^13,63,64^. Given our pulsed laser wavelength (783 nm), the corresponding CW excitation would be ∼649.5 nm. Hence, we use a 632.8 nm He-Ne source, accounting for the small residual wavelength mismatch by scaling the phonon frequency as $\frac{{\mathrm{dPos}}(2{\mathrm{D}})}{{\mathrm{d}\nu}_{\mathrm{laser}}} = 0.0132/c$ ^13^.

### Pulsed raman measurements

The ps-Raman apparatus is based on a mode-locked Er:fiber amplified laser at ∼1550 nm, producing 90fs pulses at a repetition rate RR = 40 MHz. Using second-harmonic generation in a 1 cm periodically poled Lithium Niobate crystal^65^, we obtain 3 ps pulses at 783 nm with a ∼9.5 cm^−1^ bandwidth. The beam is focused on SLG through a slightly underfilled 20× objective (NA = 0.4), resulting in a focal diameter *D* = 5.7 μm. Back-scattered light is collected by the same objective, separated with a dichroic filter from the incident beam and sent to a spectrometer (with a resolution ~0.028 nm/pixel corresponding to ~1.2 cm^−1^). The overall IRF, therefore, is dominated by the additional contribution induced by the finite excitation pulse bandwidth. Hence, in order to extract the FWHM of the Raman peaks, our data are fitted convolving a Lorentzian with the spectral profile of the laser excitation.

When using ultrafast pulses, a non-linear PL is seen in SLG^8^. Such an effect is particularly intense for the S spectral range^34,66^. The S signal in Fig. 1c is obtained as the difference spectrum of two measurements with excitation frequencies offset by ∼130 cm^−1^, resulting in PL suppression. The background subtraction requires in this case a wider spectral range, at the expenses of spectrometer resolution which is reduced to ~0.13 nm/pixel, corresponding to ∼6 cm^−1^, as additional contribution to the IRF. Although this procedure allows to isolate the S Raman peaks, the resulting noise level is worse than for AS. For this reason we mostly focus on the AS features.

**Fig. 6:**
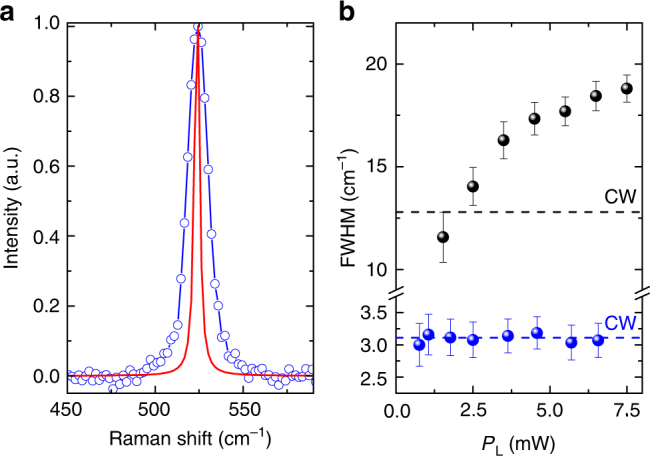
Raman response of Si for pulsed laser excitation. **a** Raman spectrum of Si measured for ultrafast laser excitation and 6.6 mW average power. (blue line) Lorentzian fit. (red line) laser bandwidth deconvoluted spectrum. **b** FWHM(Si) as a function of *P*_L_ (blue symbols) does not show any deviation from the CW FWHM(Si) (dashed blue line). FWHM(G) under the same excitation conditions (black symbols) deviates from the CW regime (dashed black line). Error bars represent the 95% confidence level of the best fit of the Si (**a**) and SLG (G band) peaks

To verify that the observed peaks broadening is not limited by our IRF, we perform the same experiment on a Si substrate (Fig. 6a). For this we retrieve, after deconvolution of the IRF, the same Raman linewidth measured in the CW excitation regime (Fig. 6a). The FWHM of the Si optical phonon is independent of *P*_L_, in contrast with the well-defined dependence on *P*_L_ observed in SLG, Fig. 6b.

### Estimate of *T*_eq_

Photoexcitation of SLG induces an excess of energy in the form of heat per unit area, *Q*, that can be expressed as:

11 $$Q \sim \frac{{P_{\mathrm{L}}}}{{\mathrm{RR}}}\frac{A}{{\pi W^2}}$$

where *A* = 2.3% is the SLG absorption, approximated to the undoped case^67^, $W \sim 2.8{\mu}{\mathrm{m}}$ is the waist of focused beam and RR = 40 MHz is the repetition rate of the excitation laser. The induced *T*_eq_ can be derived based on two assumptions: (i) in our ps timescale the energy absorbed in the focal region does not diffuse laterally, (ii) the energy is equally distributed on each degree of freedom (electrons, optical and acoustic ph). Then, *Q* can be described as:

12 $$Q = {\int}_{\mathrm{RT}}^{T_{{\mathrm{eq}}}} C(T){\kern 1pt} \mathrm{d}T$$

where *C*(*T*) is the SLG T-dependent specific heat. In the 300–700 K range, *C*(*T*) can be described as:^68^ *C*(*T*) = *aT* + *b*, where *a* = 1.35×10^−6^J K^−2^ m^−2^ and *b* = 1.35×10^−4^J K^−1^ m^−2^. Therefore, considering Eqs. 11,12, for *P*_L_ = *P*^max^ = 13.5 mW, we get $T_{{\mathrm{eq}}} \sim 680$K, well below the corresponding *T*_e_, indicating an out-of-equilibrium condition (*T*_l_ < *T*_eq_ < *T*_e_). Any contributions from the substrate and taking into account for the heat profile would contribute in reducing even further *T*_l_ estimation.

### Estimate of Pos(2D) as a function of *T*_e_

**Fig. 7:**
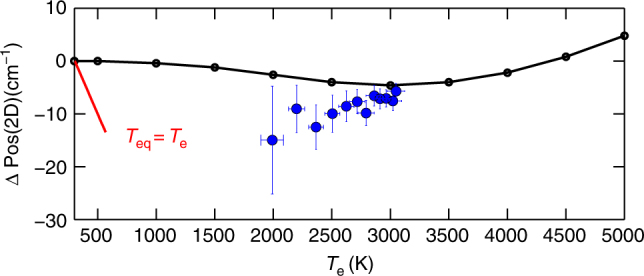
Temperature dependence of Pos(2D). Pos(2D), relative to the RT CW measurement, as a function of *T*_e_. Black line: DFPT; blue circles: experimental data with pulsed excitation. Red line: T-dependent CW measurement in thermal equilibrium (*T*_e_ = *T*_l_ = *T*_eq_) from ref.^72^. The error bars represent the 95% of confidence level, as in Fig. 3

We perform calculations within the local density approximation in DFPT^69,70^. We use the experimental lattice parameter 2.46 Å^71^ and plane waves (45 Ry cutoff), within a norm-conserving pseudopotential approach^70^. The electronic levels are occupied with a finite fictitious *T*_e_ with a Fermi–Dirac distribution, and we sample a Brillouin Zone with a 160 × 160 × 1 mesh. This does not take into account anharmonic effects, assuming *T*_l_ = 300K. Figure 7 shows a weak ΔPos(2D) ∼5 cm^−1^ in the range *T*_e_ = 300–3000 K. In equilibrium, *T*_l_ = *T*_e_ would induce a non-negligible anharmonicity^72^, which would lead to a Pos(2D) softening: ΔPos(2D)/Δ*T*_eq_ ≈ −0.05 cm^−1^ K^−1^. The weak dependence ΔPos(2D)(*P*_L_) (blue circles in Fig. 7) rules out a dominant anharmonicity contribution and, consequently, *T*_l_ = *T*_e_. The minor disagreement with DFPT suggests a *T*_l_ slightly larger than RT, but definitely smaller than *T*_eq_.

### Data availability

All relevant data are available from the authors.
